# Geriatric Assessment in Older Patients with Acute Myeloid Leukemia

**DOI:** 10.3390/cancers10070225

**Published:** 2018-07-06

**Authors:** Kah Poh Loh, Heidi D. Klepin

**Affiliations:** 1 James P. Wilmot Cancer Institute, University of Rochester Medical Center, 601 Elmwood Avenue, Box 704, Rochester, NY 14642, USA; 2 Wake Forest Baptist Comprehensive Cancer Center, Medical Center Blvd, Winston-Salem, NC 27157, USA; hklepin@wakehealth.edu

**Keywords:** comprehensive geriatric assessment, older adults, acute myeloid leukemia

## Abstract

The incidence of acute myeloid leukemia (AML) increases with age, but the outcomes for older adults with AML are poor due to underlying tumor biology, poor tolerance to aggressive treatment, and the physiologic changes of aging. Because of the underlying heterogeneity in health status, treatment decisions are difficult in this population. A geriatric assessment (GA) refers to the use of various validated tools to assess domains that are important in older adults including physical function, cognition, comorbidities, polypharmacy, social support, and nutritional status. In older patients with cancer, a GA can guide treatment decision-making, predict treatment toxicity, and guide supportive care interventions. Compared to solids tumors, there is a relative lack of studies evaluating the use of a GA in older patients with AML. In this review, we will discuss the principles, common domains, feasibility, and benefits of GA, with a focus on older patients with AML that includes practical applications for clinical management.

## 1. Introduction

Acute myeloid leukemia (AML) is the most common type of acute leukemia in adults. The incidence of AML increases with age [1]. According to the Surveillance, Epidemiology, and End Results Program, over 57% of new cases of AML occur in adults aged 65 years or above (65–74 years: 23.7%; 75–84 years: 22.8%; and >84 years: 10.6%) [1]. The outcomes of older adults with AML, however, are generally poor, with a two-year overall survival of less than 20% [2]. Contributors to poor outcomes include tumor biology, poor tolerance to aggressive treatment, and the physiologic changes of aging, including a higher prevalence of comorbidity and impairment in physical and cognitive function.

Upfront treatment for adults with AML without significant comorbidities typically consists of intensive chemotherapy with an anthracycline-containing regimen. Intensive chemotherapy in older adults is associated with treatment-related mortality of 10–20% and typically requires a prolonged period of hospitalization with significant treatment-associated disability [3,4,5]. For those who are not considered candidates for intensive chemotherapy, outpatient regimens such as hypomethylating agents (HMA) are considered [6,7,8]. Because of the heterogeneity of the underlying health of older adults with AML, determining which patients are candidates for aggressive treatments is not straightforward, making treatment decisions difficult in this population. Similarly, regardless of treatment intensity, predicting treatment complications and providing optimal supportive care planning is challenging.

Pre-treatment geriatric assessment is a strategy that can evaluate the underlying health status of older adults. In oncological settings, a geriatric assessment can assist treatment decision-making, predict treatment toxicity, and guide supportive care interventions. In this review, we will discuss the principles, common domains, feasibility, and benefits of geriatric assessment, highlighting studies in the general cancer population and specifically in older patients with AML.

## 2. Geriatric Assessment

Geriatric assessment typically consists of the use of validated tools that provide a comprehensive review of older patients’ underlying health status, and the common domains evaluated include physical function, cognition, comorbidities, polypharmacy, social support, and nutritional status. Some examples of tools used in studies of older patients with AML to assess the various domains are shown in Table 1. Symptoms and quality of life assessments have occasionally been included, but these are not typically considered as domains in geriatric assessment. The value of a geriatric assessment has been evaluated and validated in both the geriatric and geriatric oncology populations to be predictive of outcomes such as treatment toxicity, morbidity, and mortality [9,10].

## 3. Feasibility of Geriatric Assessment

In busy oncology clinics, the feasibility of performing a traditional comprehensive geriatric assessment is a persistent concern. In geriatric clinics, the time required to perform a multi-disciplinary comprehensive geriatric assessment ranges from 60–120 min [19]. It is therefore unrealistic to expect oncologists to incorporate comprehensive multi-disciplinary geriatric assessments into their routine clinical practices. In response, cancer-specific geriatric assessment tools were developed with the goal of utilizing these at the point of care in varied oncology settings [20]. A number of studies have since tested geriatric assessment in cancer populations, both in academic cancer centers and community oncology clinics [20,21,22]. The time required to complete a cancer-specific geriatric assessment reported in these studies generally ranged from 15–30 min [20,21,22]. Most assessments use self-administered validated surveys to assess domains such as physical function, emotional health, nutritional status, and social support and may incorporate brief administered tests such as a cognition screen or objectively measured physical function testing.

In the AML setting, the feasibility of performing a geriatric assessment, including comorbidity, physical function (self-reported and objectively measured), cognition, and emotional status in the inpatient setting prior to intensive induction has been demonstrated [22]. In a small prospective study, among 54 older patients who were scheduled to receive induction chemotherapy, the mean time to completion was 44.0 min (SD 14 min), and 92.6% completed the entire assessment battery. A nurse performed all objective assessments. Based on these data, a geriatric assessment was evaluated as part of a multicenter cooperative group trial (Alliance 361006) in older patients with AML prior to inpatient intensive chemotherapy [12]. This study tested a geriatric assessment developed for use in a multi-site setting. Feasibility was assessed by evaluating time to completion for the patient (comorbidity, health, medications, physical function, falls, emotional status, and social support) and healthcare professional (comorbidity, performance status, physical function, nutrition, and cognition) portions. The median time to completion was 30 min (5–65 min). Median times to completion for healthcare professionals and patients were 10 and 21 min, respectively. None of the healthcare professionals had difficulty with the assessment. Among the patients, 84% had no difficulty understanding the questions, 78% had no requirement for any assistance completing the assessment, and 82% were satisfied with the assessment length. In the pre-transplant setting, Muffly and colleagues also evaluated the feasibility of a geriatric assessment (functional status, frailty, comorbidity, emotional status, nutritional status, and degree of inflammation); 48% of the patients had AML [18]. The median time to completion was 20 min (range 15–27). Overall, these studies are consistent with those in other cancer settings, which have shown that it is feasible to incorporate a geriatric assessment into clinical trials and practice. Readers are encouraged to refer to a previously published paper for practical approaches to geriatric assessment in oncology [23]. Refinement of measures based on data from ongoing studies will help further decrease the time required and optimize integration into the clinical workflow. As mentioned above, most of the assessments are self-reported by the patients. For the objective assessments, any member of the healthcare team (most commonly a nurse or patient care technician) may perform the assessments with minimal training needed, depending on the resources available at the institution.

## 4. Benefits of Geriatric Assessment

Performing a geriatric assessment provides several benefits. First, a geriatric assessment can identify underlying impairments that are often undetected in routine oncological evaluation. In the largest prospective trial consisting of 1967 older patients with cancer (12.8% had hematologic malignancies), a geriatric assessment detected at least one geriatric problem in 51.2% of patients that would have gone undetected otherwise. Of these patients, 40.1% had functional impairment, 37.6% had nutritional impairment, 36.6% had fatigue, 30.5% had previous falls, 27.2% had depression, 19.0% had cognitive impairment, and 10.2% had social problems [24]. Other studies have demonstrated similar high rates of impairment in each of these domains [25,26,27].

Second, geriatric assessment can predict outcomes such as chemotherapy toxicity, treatment interruptions, hospitalizations, and survival. Multiple studies in various cancer subtypes have also shown predictive and prognostic values of geriatric assessment in various setting [11,28,29,30,31,32]. Two large cohort studies used geriatric assessment to develop risk prediction scores to predict chemotherapy toxicity. The Cancer and Aging Research Group (CARG) chemotherapy toxicity tool and the Chemotherapy Risk Assessment Scale for High-Age Patients (CRASH) [10,33] are both available online, although neither specifically addressed the AML setting. The CARG toxicity tool was developed and validated in older patients with solid tumors to predict grade 3–5 chemotherapy toxicity [10,33]. The CRASH score was developed in older patients with solid tumors and hematologic malignancies to predict grade 4 hematologic toxicity and grade 3 and 4 non-hematologic toxicity [34]. Both provide examples of how the information gained from geriatric assessment can be utilized efficiently to better characterize the heterogeneity of older adults of similar chronologic age.

Third, impairments detected using geriatric assessment may guide decision-making, as illustrated by the ESOGIA study, the first phase III randomized therapeutic trial guided by a geriatric assessment [35,36]. In this study, the investigators randomized older patients with stage IV lung cancer to a standard arm, in which treatment (doublet versus single agent chemotherapy) was guided by age and performance status, or a geriatric assessment-guided treatment arm. Patients in the geriatric assessment-guided arm were divided into three groups (fit, vulnerable, and frail) based on comorbidity, physical function, cognition, emotional status, and presence of geriatric syndromes. Fit older patients received doublet chemotherapy, whereas vulnerable patients received single agent chemotherapy, and frail patients received best supportive care. Although no overall survival difference was found, in the geriatric assessment-guided arm, more fit patients received doublet chemotherapy and frail patients were spared chemotherapy toxicity [35,36]. In a separate study, Tucci and colleagues risk-categorized 177 older patients with diffuse large B-cell lymphoma to fit and unfit based on their age, comorbidity, and physical function [37]. The decision to treat and choice of treatment were left to the discretion of their treating physicians. They found that patients who were fit benefited from treatment with curative intent compared to treatment with palliative intent, and the unfit group derived the same benefits whether they received treatment with palliative or curative intent. Both aforementioned studies demonstrated that a geriatric assessment could be helpful in guiding treatments. When relevant geriatric information is available to oncologists, it has been shown that treatment decision-making does change [38,39]. While geriatric assessment-guided AML studies are lacking, available data from varied settings do suggest that if geriatric assessment information is available to oncologists, decisions to treat more or less aggressively are altered; some patients receive more aggressive treatment based on evidence of fitness and others receive less aggressive therapies once existing vulnerabilities or frailty are demonstrated.

Fourth, geriatric assessment can guide management interventions in response to the impairment identified [40]. In a multicenter prospective study, 35.5% of the 1550 patients who received a geriatric assessment subsequently received management interventions with referral to a dietician (60.4%) being the most common, followed by referral to a social worker (40.3%), and referral to a psychologist (28.9%) [41]. Nonetheless, the impact of geriatric assessment-guided management is still an active area of investigation. In a comparative study of two cohorts of older patients on chemotherapy, those who received geriatric assessment-guided management were found more likely to complete cancer treatment and required fewer treatment modifications [42]. On the other hand, a randomized pilot study did not demonstrate any difference in treatment outcomes and toxicities [43]. The difference in findings is likely due to the varying sample size (135 in the observational cohort study versus 71 patients in the randomized pilot study) and specific outcomes assessed. In the observational cohort study, outcomes were completion of treatment and dose interruptions, whereas in the randomized pilot study, the outcomes were dose reduction, delays, and early termination. Nevertheless, published studies to date support the feasibility of geriatric assessment-guided management [42,43], which will be an important area of investigation for older adults with AML.

## 5. Emerging Role of Geriatric Assessment in Acute Myeloid Leukemia

The utility of geriatric assessment in older patients with AML in detecting underlying impairments and predicting outcomes, namely in the upfront setting and prior to allogeneic stem cell transplant, is increasingly being studied (Table 1). In the upfront setting, Klepin and colleagues assessed geriatric domains in 74 older patients who were scheduled to receive intensive induction chemotherapy in a prospective trial [11]. Among these patients, 41.9% had comorbidity, 40.5% to 72.4% had functional impairments (self-reported or objectively measured), 28.8% had cognitive impairments, 39.7% had depression, and 58.9% had distress. It is worth mentioning that 20.6% to 41.1% of these patients recalled having functional impairment 6 months prior. After induction chemotherapy, distress improved, but functional impairment worsened [4]. In multivariable analysis, after being adjusted for age, gender, performance status, cytogenetics, prior myelodysplastic syndrome (MDS), and hemoglobin level, mortality was higher in patients with impaired objective physical function (measured using the Short Physical Performance Battery (SPPB, comprised of gait speed, repeat chair stands, and balance testing); Hazard ratio (HR) 2.2, 95% Confidence Interval (CI) 1.1–4.6) and impaired cognition (measured using the modified Mini-Mental State Examination (3MS); HR 2.5, 95% CI 1.2–5.5) compared to those who did not have these impairments at baseline [11]. Interestingly, functional impairment as recalled by patients themselves 6 months prior to diagnosis was not predictive of survival further supporting the importance of assessment at the time of treatment decision-making.

In a second study, Deschlar and colleagues performed a geriatric assessment in all older patients with newly diagnosed AML (n = 132) and MDS (n = 63), irrespective of treatment received, seen at two institutions over 2 to 4 years [13]. Among these patients, 55.0% had functional impairment, 8.7% had cognitive impairment, and 14.3% had depression. More impairment was noted in patients who received low intensity therapy and best supportive care, compared to intensive regimens. In multivariable analysis, after adjusting for clinical and disease features such as performance status, bone marrow blast count, and cytogenetics, mortality was higher in those with comorbidity (measured using the Hematopoietic cell transplantation-comorbidity index (HCT-CI); HR 2.0, 95% CI 1.1–3.4), impaired activities of daily living (measured using the Barthel Index; HR 2.5, 95% CI 1.4–1.9), and self-reported fatigue (measured using the European Organization for Research and Treatment of Cancer (EORTC) QLQ-C30; HR 1,8, 95% CI 1.0–3.2). A third retrospective study also demonstrated that comorbidity (HCT-CI), pain (EORTC QLQ-C30), and self-reported difficulty with strenuous activity (EORTC QLQ-C30) are prognostic in this population [14].

In addition to the aforementioned studies, individual geriatric domains within the geriatric assessment, such as polypharmacy, cognitive impairment, comorbidity, and physical function have been assessed in various studies that included older patients with newly diagnosed AML and are associated with worse outcomes [15,16,44]. For example, older patients with AML who were on ≥4 medications (versus ≤1) had higher 30-day mortality (Odds Ratio (OR) 9.98, 95% CI 1.2–84.1), lower odds of complete remission status (OR 0.2, 95% CI 0.1–0.7), and higher overall mortality (HR 2.1, 95% CI 1.2–4.0) [16]. In older patients with hematologic malignancies including AML, those with impairment in working memory had worse survival (OR 0.3 95% CI, 0.1–0.5); median overall survival was 10.9 (standard deviation (SD) 12.9) for those with impairment versus 12.2 (SD 14.7) months without impairment (*p* < 0.01) [44]. In terms of comorbidity, increasing numbers of comorbidities and specific comorbidities (such as diabetes) are also associated with worse outcomes [17,45,46]. Poor physical function, as assessed using the instrumental activities of daily living, was associated with higher mortality even after adjustment for cytogenetics and performance status (HR 4.3, 95% CI 1.7–10.5) [15]. In the pre-transplant setting, Muffly and colleagues also demonstrated that overall survival was worse in older patients (48% AML) with impaired instrumental activities of daily living (HR 2.4, 95% CI 1.6–3.6), slow walk speed (HR 1.8, 95% CI 1.1–2.8), high comorbidity (HR 1.56, 95% CI 1.1–2.3), and impaired emotional status (HR 1.7, 95% CI 1.1–2.5) [18].

By detecting impairment in older patients with AML, these findings may inform upfront treatment allocation, select candidates for hematopoietic stem cell transplantation, and guide supportive care management. Nevertheless, geriatric assessment-guided treatment allocation is evolving and remains an active area of investigation [47,48]. In terms of supportive care management, interventions such as exercise programs to improve physical function, symptoms, and emotional status, and psychosocial interventions to improve emotional status may be helpful in older patients with AML [49,50].

## 6. Geriatric Screening Tools

In other cancer subtypes, the use of geriatric screening tools may spare the effort of a geriatric assessment in approximately 30% of patients [24]. These screening tools include Geriatric 8 (G8), Vulnerable Elders Survey-13, Triage Risk Screening Tool (TRST), Groningen Frailty Index, Senior, Adult Oncology Program 2, Abbreviated GA, and Fried frailty criteria [41,51,52,53,54,55]. These screening tools may be used to detect potential vulnerabilities and guide the need for a geriatric assessment in older patients with AML. However, none of these tools have been evaluated solely in the AML population. Several studies evaluating G8 have included variable numbers of patients with hematologic malignancies (some had more patients with AML than others), and may be considered for use if a geriatric assessment cannot be performed [51]. The G8 consists of 8 questions and it screens for comorbidity, polypharmacy, functional status, cognition, and nutritional status, in addition to chronologic age and self-perceived health status [55]. Median time to completion was 4 min [56]. 

## 7. Ongoing and Future Studies

Many gaps remain in our knowledge of how best to utilize geriatric assessment in the management of older patients with AML. First, we need to incorporate geriatric assessment in therapeutic studies of older patients with AML in order to better understand the underlying health status of patients enrolled in clinical trials and help us predict treatment toxicity and risk stratify patients. In this regard, the Southwest Oncology Group (SWOG) trial S1612 (NCT03092674) that evaluates novel therapies in older patients with AML is currently ongoing with planned prospective collection of geriatric assessment information [57]. There have also been efforts to incorporate geriatric assessment into transplant decision-making and chemotherapy toxicity prediction. By identifying patient characteristics that influence risk of toxicity or predict resilience, we can apply scientific rigor to the characterization of fitness or frailty in a specific treatment setting. This is important for decision-making and can inform trial design by providing information to support novel subset selection, evidence-based eligibility criteria, and statistical designs which simultaneously evaluate the implications of both tumor biology and patient heterogeneity on treatment outcomes (personalized medicine). Second, in addition to predicting outcomes, we need to study whether geriatric assessment-guided management can improve outcomes. Geriatric assessment-guided management can involve targeting identified modifiable vulnerabilities to improve treatment tolerance (such as intervening on physical or emotional health) or personalizing treatment regimens based on geriatric assessment profile. In particular, outcomes such as treatment interruptions, completion, and toxicity as well as patient-reported outcomes such as quality of life should be assessed. Finally, geriatric assessment measures, which rigorously capture the impact of treatment on critical domains such as physical, emotional, and cognitive health, should be incorporated into trial design to complement global QOL measures and PRO-based symptom assessment and provide additional evidence to support the benefits of any given therapeutic approach.

## 8. Conclusions

In summary, older patients with AML are heterogeneous in their health status. Geriatric assessment may be used to detect impairment, predict outcomes, assist in treatment decision-making, guide supportive care interventions, and potentially improve outcomes in this vulnerable population.

## Figures and Tables

**Table 1:** Geriatric domains assessed and tools used in studies of older patients with acute myeloid leukemia.

Study	Study Sample	N (Mean or Median Age If Available)	Design	Domains	Tools Used
Klepin, et al. 2013 [11]	Newly diagnosed AML, intensive chemotherapy	74 (Mean age = 70)	Single-center, prospective	Comorbidity	HCT-CI
				Functional status	PAT-D, SPPB, grip strength
				Cognition	3MS
				Depression	CES-D
				Distress	Distress Thermometer
Klepin, et al. 2014 [12]	Newly diagnosed AML, intensive chemotherapy	43 (Median age = 67.6)	Multi-center, prospective	Comorbidity	HCT-CI, OARS Physical Health Subscale
				Functional status	Timed up and go test, ADL, IADL, Falls, KPS
				Cognition	BOMC
				Depression	MHI-17
				Nutrition	BMI, % weight loss in the past 6 months
				Social Support	MOS Social activity limitations/social support subscales
Deschlar, et al. 2013 [13]	Newly diagnosed AML and MDS, any treatment	195 (Median age = 71)	Multi-center, retrospective	Comorbidity	HCT-CI, CCI
				Functional status	KPS, ADL, IADL, Timed up and go test
				Cognition	MMSE
				Depression	GDS-15
				Quality of life	EORTC QLQ-C30
Sherman, et al. 2013 [14]	Newly diagnosed AML	101	Single-center, retrospective	Comorbidity	HCT-CI
				Quality of life (physical, social and cognitive functioning, psychological status, nutritional status, and pain)	EORTC QLQ-C30
Wedding, et al. 2006 [15]	Newly diagnosed AML, any treatment	63 (Median age = 61.1)	Single-center, prospective	Functional status	KPS, IADL
Elliot, et al. 2014 [16]	Newly diagnosed AML, intensive chemotherapy	150 (Median age = 69)	Single-center, retrospective	Polypharmacy	Number of medications
Tawfik, et al. 2016 [17]	Newly diagnosed AML, intensive chemotherapy	144 (Median age = 70)	Single-center, retrospective	Comorbidity	CCI
Muffly, et al. 2014 [18]	Patient scheduled to undergo allogeneic HSCT	203 (Median age = 58)	Single-center, retrospective	Comorbidity	HCT-CI, CIRS-G
				Functional status	ADL, IADL, SF36-PCS, ECOG PS, grip strength, walk speed
				Mental health	SF36-MCS
				Frailty	Fried Frailty Index
				Nutrition	Weight loss

HCT-CI: Hematopoietic Cell Transplant-Comorbidity Index; PAT-D: Pepper Assessment Tool for Disability; SPPB: Short Physical Performance Battery; 3MS: Modified Mini-Mental state examination; CES-D: Center for Epidemiological Studies-Depression; OARS: Older Americans Resources and Services; ADL: activities of daily living; IADL: instrumental activities of daily living; KPS: Karnofsky performance status; BOMC: Blessed Orientation-Memory-Concentration; MHI-17: Mental Health Inventory-17; BMI: body mass index; MOS: Medical Outcomes Study; CCI: Charlson Comorbidity Index; MMSE: Mini-Mental State examination; GDS-15: Geriatric Depression Scale-15; EORTC QLQ-C30: European Organisation for Research on Cancer Treatment Quality of Life Questionnaire; CIRS-G: Cumulative Illness Rating Scale-Geriatric; SF36-PCS: Medical Outcomes Short Form-36 health-related quality of life questionnaire; ECOG PS: Eastern Cooperative Oncology Group performance status; SF36-MCS: Medical Outcomes Short Form-36 health-related quality of life questionnaire-mental component score.

## Abbreviations

3MS	Modified Mini-Mental state examination
ADL	activities of daily living
AML	acute myeloid leukemia
BOMC	Blessed Orientation-Memory-Concentration
BMI	body mass index
CCI	Charlson Comorbidity Index
CES-D	Center for Epidemiological Studies-Depression
CIRS-G	Cumulative Illness Rating Scale-Geriatric
ECOG	Eastern Cooperative Oncology Group
HCT-CI	Hematopoietic Cell Transplant-Comorbidity Index
IADL	instrumental activities of daily living
EORTC QLQ-C30	European Organisation for Research on Cancer Treatment Quality of Life Questionnaire
GDS	Geriatric Depression Scale
KPS	Karnofsky performance status
HSCT	hematopoetic stem cell transplantation
MCS	mental component score
MHI-17	Mental Health Inventory-17
MMSE	Mini-Mental State examination
OARS	Older Americans Resources and Services
PAT-D	Pepper Assessment Tool for Disability
PS	performance status
SF-36	Medical Outcomes Short Form-36 health-related quality of life questionnaire
MOS	Medical Outcomes Study
